# IgG Study of Blood Sera of Patients with COVID-19

**DOI:** 10.3390/pathogens10111421

**Published:** 2021-11-02

**Authors:** Elena Kazachinskaia, Alexander Chepurnov, Dmitry Shcherbakov, Yulia Kononova, Teresa Saroyan, Marina Gulyaeva, Daniil Shanshin, Valeriya Romanova, Olga Khripko, Michail Voevoda, Alexander Shestopalov

**Affiliations:** 1 Federal Research Center of Fundamental and Translational Medicine, The Federal State Budget Scientific Institution, Siberian Branch of the Russian Academy of Sciences (SB RAS), 630117 Novosibirsk, Russia; lena.kazachinskaia@mail.ru (E.K.); alexa.che.purnov@gmail.com (A.C.); yuliakononova07@yandex.ru (Y.K.); 111.st.13@rambler.ru (T.S.); valeriaromanovavr@mail.ru (V.R.); khripkoolga@gmail.com (O.K.); mvoevoda@ya.ru (M.V.); shestopalov2@mail.ru (A.S.); 2 State Research Centre of Virology and Biotechnology “VECTOR”, Federal Service for Surveillance in the Sphere, Consumers Rights Protection and Human Welfare, 630117 Novosibirsk, Russia; scherbakov_dn@vector.nsc.ru (D.S.); shanshin_dv@vector.nsc.ru (D.S.); 3 The Department of Naturel Science, Novosibirsk State University, 630090 Novosibirsk, Russia

**Keywords:** SARS-CoV-2, COVID-19, antibody specificity, recombinant proteins, virus neutralization

## Abstract

The COVID-19 pandemic, which began at the end of 2019 in Wuhan, has affected 220 countries and territories to date. In the present study, we studied humoral immunity in samples of the blood sera of COVID-19 convalescents of varying severity and patients who died due to this infection, using native SARS-CoV-2 and its individual recombinant proteins. The cross-reactivity with SARS-CoV (2002) was also assessed. We used infectious and inactivated SARS-CoV-2/human/RUS/Nsk-FRCFTM-1/2020 strain, inactivated SARS-CoV strain (strain Frankfurt 1, 2002), recombinant proteins, and blood sera of patients diagnosed with COVID-19. The blood sera from patients were analyzed by the Virus Neutralization test, Immunoblotting, and ELISA. The median values and mean ± SD of titers of specific and cross-reactive antibodies in blood sera tested in ELISA were mainly distributed in the following descending order: N > trimer S > RBD. ELISA and immunoblotting revealed a high cross-activity of antibodies specific to SARS-CoV-2 with the SARS-CoV antigen (2002), mainly with the N protein. The presence of antibodies specific to RBD corresponds with the data on the neutralizing activity of blood sera. According to the neutralization test in a number of cases, higher levels of antibodies that neutralize SARS-CoV-2 were detected in blood serum taken from patients several days before their death than in convalescents with a ranging disease severity. This high level of neutralizing antibodies specific to SARS-CoV-2 in the blood sera of patients who subsequently died in hospital from COVID-19 requires a thorough study of the role of humoral immunity as well as comorbidity and other factors affecting the humoral response in this disease.

## 1. Introduction

According to the WHO, the COVID-19 pandemic, caused by a new pathogen SARS-CoV-2 in the human population, which began at the end of 2019 in Wuhan, in China’s Hubei province, has affected 220 countries and territories to date. As of 29 June 2021, the total number of reported cases worldwide exceeds 180 million, and the number of deaths worldwide is almost 4 million [1]. In many countries, quarantines and other milder strategies for preventing the spread of the infection, such as physical distancing and the use of masks, may have prevented most people from being infected. According to the opinion of some researchers, this is a paradox, as such measures leave people without immunity, susceptible to new waves of infection. Healthcare workers, the elderly, and people with medical conditions, such as cardiovascular and cerebrovascular diseases, diabetes, and neoplasms, are at a particularly high risk of infection [2,3]. It is quite possible that the modern world will not return to “pre-pandemic normality” until safe and effective vaccines have been developed and a global vaccination program has been implemented [4]. 

The latest reports show that most COVID-19 patients develop lymphopenia as well as pneumonia, with higher plasma levels of pro-inflammatory cytokines in severe cases, suggesting that the host immune system is involved in the pathogenesis [5]. However, there is still a very limited understanding of the immune responses, especially adaptive immune responses to SARS-CoV-2 infection. Since COVID-19 is a new disease for humanity, and the nature of the protective immune responses against it is poorly understood, it is also unclear what vaccination strategies will be the safest and most immunologically successful. It is quite possible that vaccine-induced protection may differ from natural immunity due to the virus’s different evading strategies [6] and/or due to acquired humoral immunopathology in the form of antibody-dependent enhancement of infection and/or antigenic imprinting [7]. It is important to understand adaptive immunity to SARS-CoV-2, not only for vaccine development, but for interpreting COVID-19 pathogenesis and the calibration of pandemic control measures. 

In this regard, data from the studies of the natural immune response to COVID-19 will contribute to COVID-19’s pathogenesis, the development of effective vaccines, and therapeutic strategies. It is especially important to make clear the difference in immune responses between asymptomatic, mild, and severe cases and in the early and late stages of infection. In addition, the fact that asymptomatic and mildly suffering people develop a low level of antibody-mediated protection is important for assessing herd immunity [6]. At this moment, in the territory of Russia, this topic is not highlighted fully enough. Our investigation relates to the study of the immune response in patients with a different course of COVID-19 at the very beginning of the pandemic in one of the biggest cities of Russia under restrictions (the absence of air and railway connections with neighboring countries). It may reflect the situation at a specific point and is valuable precisely in a retrospective analysis of the development of the epidemic process in comparison with both other regions of Russia and other countries. In detail, the aim of this study was to examine humoral immunity in the samples of convalescents’ blood sera with COVID-19 in a range of severities, and patients who subsequently died in hospital from this disease, using native SARS-CoV-2 and its individual recombinant proteins to visualize an individual immune "picture" of antibodies, i.e., their profile for specificity to the N proteins, the S trimer, and RBD. We consider the presence of such antibodies as part of the immune response to SARS-CoV-2. Previously, it was reported that sera from some patients could inhibit SARS-CoV-2 entry in target cells, indicating the involvement of humoral immunity due to anti-S antibodies as early as three days post symptom onset. Protein N is a major immunogen, and antibodies to it are formed in some patients who have been ill [5]. The antibodies to protein M are observed less often, and we assume that this fact would also be interesting to study. The cross-activity of antibodies with the inactivated SARS-CoV antigen (2002) was also assessed.

## 2. Results

To study humoral immunity against SARS-CoV-2, a random sample of 54 blood sera received from 26 convalescents (1 asymptomatic case, 13 mild cases, 1 moderate case with hospitalization for pneumonia and 1 without hospitalization, 10 severe cases, and 13 severe cases with a lethal outcome in the hospital) was used. In some cases, serum was paired from one patient. The titers of specific interaction of antibodies with these antigens were found in blood sera by the author’s laboratory ELISA, using inactivated whole-cell SARS-CoV-2/human/RUS/Nsk-FRCFTM-1/2020 and SARS-CoV (strain Frankfurt 1, 2002) preparations as antigens, as well as the SARS-CoV-2 recombinant proteins—the N nucleoprotein, the full S trimer (spike) and RBD, its receptor-binding domain in the S1 region. For recombinant proteins, preparation the plasmids for the expression of the full N and full S trimer (spike) and also its part in the S1 region, RBD, were constructed with further purification of obtained proteins. 

### 2.1. The Blood Sera Study of COVID-19 Convalescents of Varying Severity with a Favorable Outcome

**Figure A1:**
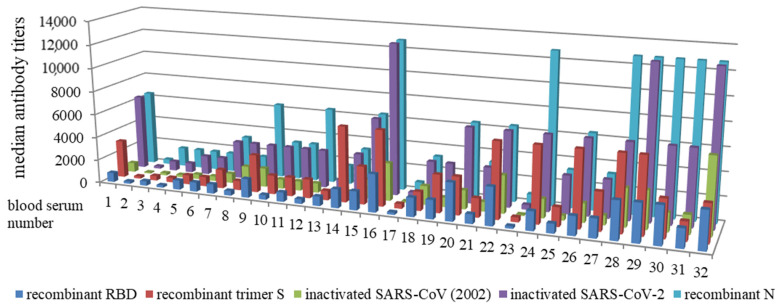
Specific interaction of IgG convalescents with inactivated viral preparations and recombinant proteins in ELISA. Note. Blood sera: No. 1—an asymptomatic case; No. 2–14—mild cases, No. 15—moderate case with unilateral pneumonia; No. 16—moderate case with hospitalization for bilateral pneumonia; No.17–32—severe cases of patients who were treated in the intensive care unit. Data on the neutralizing activity of these blood sera are presented in Table S1 and Table S2.

During our study, it turned out that in mild cases, the median values of titers of IgG specific to the inactivated SARS-CoV-2 antigen were in the range of 1:200–1:12,800, with the most common values being 1:800 and 1:3200. High titers were found in an asymptomatic patient as well as in the case of a patient who recovered after a moderate case of COVID-19 (with unilateral pneumonia)—1:6400 (Numbers 1 and 15 on the Appendix A Figure A1). Three more recovered cases with bilateral pneumonia, or those who were treated in the intensive care unit, showed the highest titers—1:12,800, in the neutralization test with the inactivated SARS-CoV-2 antigen (Appendix A Figure A1).

The cross-activity of antibodies specific to SARS-CoV-2 with the inactivated heterologous SARS-CoV (strain Frankfurt 1, 2002) antigen (the third row on the Appendix A Figure A1, marked in green) increased in accordance with the titer found on the SARS-CoV-2 homologous antigen.

The use of recombinant SARS-CoV-2 proteins in ELISA allowed us to determine the individual profile of antibodies specific to individual viral proteins. According to ELISA data in median values (Appendix A Figure A1) and the mean ± SD of titers of specific antibodies (Table S1), the antibodies in this panel of convalescents’ blood sera, from mild to severe cases in specificity and quantitatively, are mainly distributed in the following descending order: N > S trimer > RBD. 

Higher IgG levels with median values in the range of 1:400 to 1:3200, detected in ELISA using the recombinant RBD protein (Appendix A Figure A1), contributed to more effective protection of infected Vero cells from the development of a cytopathic effect by 50%—from 1:20 to 1:160 in mild cases (Numbers 3, 5, 6–14), and from 1:80 to 1:≥320 in cases of moderate severity (Numbers 15, 16) and seriously ill patients (Numbers 18–32). In an asymptomatic patient (Number 1, age 32), the titer of protective antibodies was 1:80 (data on neutralization) (Table S2).

In the presence of paired sera from patients with severe cases of COVID-19 (Numbers 17–28), an increase in IgG titers specific to RBD in ELISA was observed (Appendix A Figure A1).

Obviously, the presence of antibodies specific to RBD correlates with the data on the neutralizing activity of blood sera. So, for example, the 1:200 titer of IgG specific to this site of the S glycoprotein detected in ELISA either did not contribute to protection in a severe case (Numbers 17 and 23) or corresponded to the presence of neutralizing antibodies in a titer of 1:10–1:20 in the mild cases of Numbers 2 and 4 (Table S2). In patients with a severe course of the disease from whom only single serum samples were studied in the neutralization reaction (Numbers 29–32), the median titers of protective antibodies turned out to be 1:160. In the presence of paired blood sera in patients with a severe course of the disease, a tendency towards a sharp increase in the titers of protective antibodies was revealed within a few days from complete absence to 1:160 (Numbers 23, 24) or to 1:≥320 (Numbers 17, 18). In other patients with a severe course of the disease, the titers of protective antibodies also steadily increased—for example, from 1:160 to 1:≥320 (Numbers 19, 20 and 27, 28) and from 1:80 to 1:160 (Numbers 21, 22 and 25, 26) over the course of several days, respectively (Table S2).

**Figure A2:**
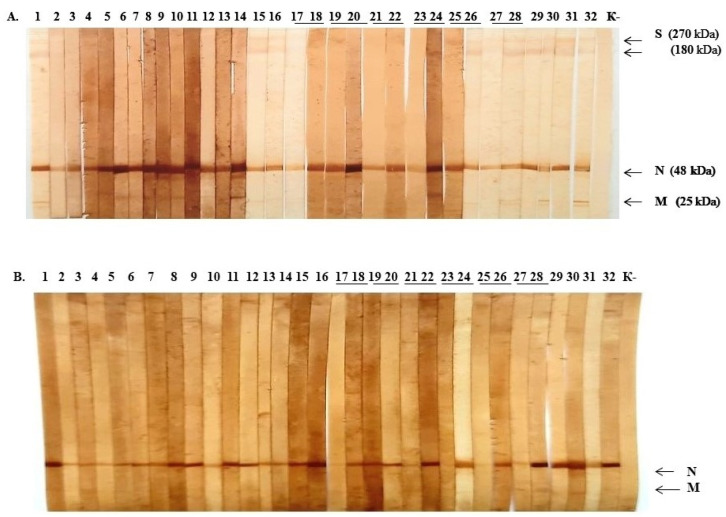
Immunoblotting of viral proteins of the SARS-CoV-2/human/RUS/Nsk-FRCFTM-1/2020 (2020) and SARS-CoV (2002) strains with antibodies of convalescents’ blood sera (asymptomatic, mild or moderate cases of COVID-19, as well as patients in the intensive care unit). (**A**). The SARS-CoV-2/human/RUS/Nsk-FRCFTM-1/2020 antigen on the membrane. (**B**). The SARS-CoV antigen (2002) on the membrane. Blood sera: No. 1—an asymptomatic case; No. 2–14—mild cases, No. 15—a moderate case with unilateral pneumonia; No. 16—a moderate case with hospitalization for bilateral pneumonia; No. 17–32—severe cases of patients in the intensive care unit. K- (negative control)—blood serum of a healthy person. The numbers of paired blood sera are underlined.

The immunoblotting of the SARS-CoV-2/human/RUS/Nsk-FRCFTM-1/2020 antigen with the blood serum of an asymptomatic patient, as well as those with mild and moderate cases, made it possible to detect viral target proteins for specific antibodies. Moreover, it was also possible to visually determine the efficiency/intensity of the detection of these viral proteins in this enzyme immunoassay on the nitrocellulose membrane with antibodies used for testing the same dilution of blood serum—1:100. It was clearly demonstrated that the N nucleocapsid protein of SARS-CoV-2 is the main protein target for antibodies in mild cases. Numbers 1–32 include the antibodies of asymptomatic (Number 1), mild (Numbers 2–14), and moderate patients (Numbers 15 and 16), as well as patients from the intensive care unit (Numbers 1–32) (Appendix A Figure A2A). 

The antibodies specific to linear epitopes of the S-protein were not detected in all blood sera. For example, the SARS-CoV-2 S-protein dimer and trimer were detected in the serum of an asymptomatic patient (Number 1) at 180 and 270 kDa, respectively. Additionally, among mild cases of the disease (Numbers 2–14), possibly due to background levels, there are no clear bands of the S protein. Antibodies were also detected in those with moderate cases of COVID-19 (Numbers 15 and 16). In severe cases of the disease (intensive care unit) (Numbers 17–32), antibodies specific to linear epitopes of the S-protein were detected in the sera (Numbers 24–32). These findings strongly demonstrate that the intensity of the immune response significantly increased in paired sera—Numbers 23–24, 25–26, and 27–28.

The SARS-CoV (2002) nucleoprotein mostly turned out to be the only target protein for cross-interaction of antibodies specific to SARS-CoV-2 in asymptomatic, mild, moderate (Numbers 1–16), and severe cases (Numbers 17–32). 

In some cases, the antibodies of some blood sera detected the M protein SARS-CoV-2 (Appendix A Figure A2A). The M protein SARS-CoV (2002) was also the target of weak cross-interaction for these antibodies (Appendix A Figure A2B).

### 2.2. The Blood Sera Study of Patients with a Fatal Outcome of Disease

In our study, we used sera from 13 patients with severe cases of COVID-19 with a lethal outcome in the hospital. It was shown that according to the ELISA results, the median values of specific IgG titers in the sera of these patients ranged from 1:200 to 1:25,600 (Table S3).

**Figure A3:**
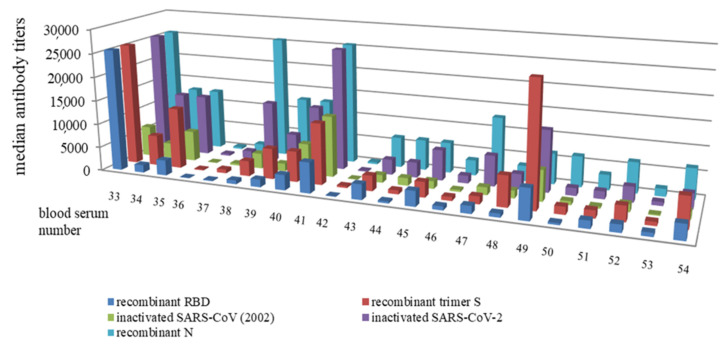
Titers of specific IgG interactions in patients with severe cases of COVID-19 who subsequently died in hospital, with inactivated viral preparations and recombinant proteins in ELISA. Note: data on blood sera numbers are presented in Table S1 and Table S3.

In the case of the paired blood sera, just as in the case of convalescents, in patients who subsequently died in the hospital, an increase in antibody titers was observed when using the inactivated SARS-CoV-2 antigen. This is especially evident in the example of three blood sera from one patient with an increase in IgG titers in ELISA from 1:6400 to 1:12,800 and 1:25,600 in blood sera for Numbers 39, 40, and 41, taken at the 15th, 10th, and 7th days before death, respectively. Moreover, these blood sera contain antibodies specific to RBD in titers of 1:1600, 1:3200, and 1:6400, corresponding to the neutralizing antibodies in the range of 1:160 to ≥1:320 a week before death (Table S3). The neutralizing antibodies at the median values of titers ≥1:320 were also found in single blood sera from two patients (Numbers 33 and 35), who subsequently died a day after the blood was taken. The sufficiently high serological titers on the inactivated antigen (1:25,600 and 1:12,800) and on the recombinant RBD (1: 25,600 and 1:3200) in ELISA were also shown (Appendix A Figure A3). 

The antibodies of the blood sera of 13 patients that died from severe COVID-19 in the hospital actively cross-interact with the SARS-CoV antigen (2002) in ELISA (Appendix A Figure A3). A dependence on the value of titers with a homologous antigen was also shown, i.e., a higher titer of antibodies to SARS-CoV-2, causing a higher value of cross-activity. 

**Figure A4:**
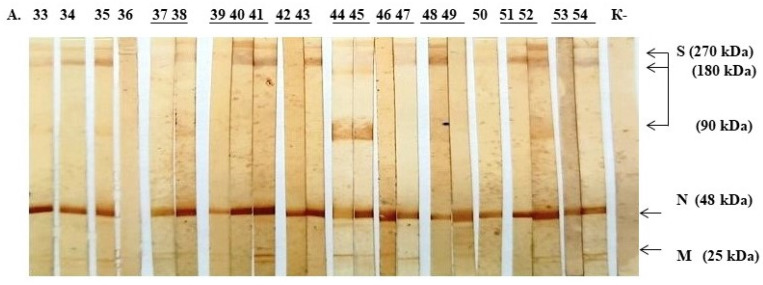

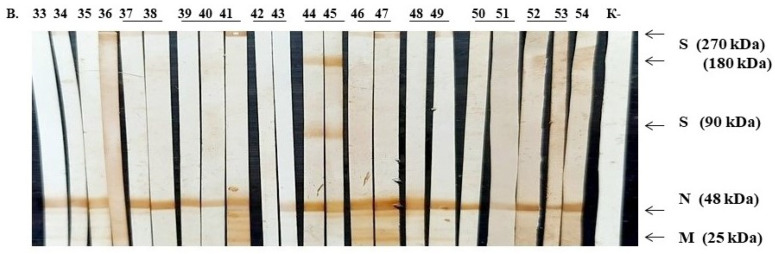
Immunoblotting of viral proteins of the SARS-CoV-2/human/RUS/Nsk-FRCFTM-1/2020 (2020) and SARS-CoV (2002) strains with antibodies of blood sera of patients who were treated in the intensive care unit diagnosed with COVID-19 (later died in the hospital). (**A**) SARS-CoV-2/human/RUS/Nsk-FRCFTM-1/2020 antigen on the membrane. (**B**) SARS-CoV antigen (2002) on the membrane. *K-* (negative control)—blood serum of a healthy person. The numbers of paired blood sera are underlined.

According to immunoblotting data (Appendix A Figure A4A), the N protein of SARS-CoV-2 is detected by antibodies of all patients in the intensive care unit (who later died in the hospital), except for the blood serum Number 36. The presence of paired blood sera taken at different times before the death of patients makes it possible to clearly demonstrate the results of ELISA (Appendix A Figure A3) on the increase in antibody titers and the presence of antibodies specific to the linear epitopes of the S protein (dimer and trimer), and, in some cases, to the M protein. In one case, antibodies specific to the linear epitopes of the S protein monomer (90 kDa) were observed (blood sera Numbers 44 and 45). The same antibodies were also cross-reactive with analogous epitopes of the S protein monomers of SARS-CoV (2002) (Appendix A Figure A4B).

As shown in Appendix A Figure A4, in many patients in the intensive care unit (who later died in the hospital), cross-reactive antibodies were specific to both the N protein and to the S protein trimer. In serum Number 36, there were antibodies that detected linear epitopes of the S protein on the SARS-CoV antigen (2002) that did not manifest themselves in the neutralization reaction with SARS-CoV- 2 and did not reveal any bands in the SARS-CoV-2 immunoblotting.

## 3. Discussion

It is a fact that SARS-CoV-2 continues to spread rapidly on a global scale. Because of this, a better understanding of the relationship between the immune response and the severity of infection is needed. Serological tests are considered to be one of the main tools for epidemiological surveillance. If reliable serological data are available, the number of deaths associated with COVID-19 can help to determine the infection fatality ratio (IFR) for the disease. This knowledge can help to establish the relationship between fatalities and the total number of infections [8]. In our work, we thus provide a basis for further analysis of the pathogenesis of COVID-19, especially in severe cases, and protective immunity to SARS-CoV-2. It may also be implicated in designing an effective vaccine to protect against and treat SARS-CoV-2 infection.

Serological tests are considered to be a powerful complement to nucleic acid tests, especially for COVID-19 patients with undetectable viral RNA. Most of these tests based on the method of immunochromatography or ELISA and recombinant SARS-CoV-2 proteins have different sensitivity and specificity. In this regard, scientific groups studying the immune response of patients to COVID-19 use both commercial diagnostic kits and kits of their own production [9]. 

We have developed an author’s ELISA test system based on antigens obtained by our scientific group. These include inactivated purified concentrated native viral preparations (SARS-CoV-2/human/RUS/Nsk-FRCFTM-1/2020 and SARS-CoV strain Frankfurt 1, 2002) and the recombinant proteins N, S, and RBD. This test system was used to determine the median titers of specific and cross-reactive antibodies in the blood sera of convalescents and patients who subsequently died from COVID-19 infection. It is shown that the cross-activity of antibodies (IgG resulting from COVID-19) with the inactivated heterologous SARS-CoV antigen (2002) increased in accordance with their titer, determined based on the homologous SARS-CoV-2 antigen in ELISA. The use of recombinant SARS-CoV-2 proteins allowed us to determine the individual profile of antibodies specific to individual viral proteins. According to the ELISA data, these antibodies in this panel of blood serum convalescents from mild to severe COVID-19, as well as in subsequent patients who died in the hospital, are distributed by specificity and quantitatively (in median titer values), mainly in the following descending order: N > trimer S > RBD. 

The immunoblotting allowed us to identify linear epitopes of the main SARS-CoV-2 target proteins. In the case of a mild case of COVID-19, it is mainly a nucleoprotein. In moderate and severe cases with a favorable outcome, these are the nucleoprotein and the S trimer (270 kDa) and dimer (180 kDa). In a severe clinical course and a fatal outcome, antibodies specific to both the nucleoprotein and the S trimer (270 kDa) and dimer (180 kDa) were found in patients at different intervals before death. The literature data confirm that these coronavirus proteins are the most immunogenic of the four structural viral proteins, in addition to E (envelope) and M (membrane), to which the humoral immune response of the infected body is formed [10]. When the antibodies of some sera from surviving patients of severe COVID-19 do not interact with the linear epitopes of the S protein in immunoblotting, this cannot indicate the absence of such antibodies. In fact, it is most likely that they may be specific to the significant conformational epitopes of this viral protein, which are destroyed by abrupt electrophoretic separation.

The SARS-CoV nucleoprotein (2002) in immunoblotting was the major protein for cross-reactive antibodies in asymptomatic, mild, moderate, or severe COVID-19 with a favorable outcome.

The antibodies of some blood sera detected the M protein, which also causes the production of specific antibodies in patients, as shown by Guan M. et al. while testing the blood sera by immunoblotting, both when using an inactivated viral preparation and recombinant M protein received in eukaryotic expression system [11]. In our previous report [12], we confirmed the data of Sturman L. et al. [13], who showed that in order to visualize the matrix protein of mouse coronavirus A59, the viral preparation should not be heated prior to the electrophoretic separation of proteins. 

Therefore, in this work, a viral preparation was used without heating, and it was found that not all patients suffering from COVID-19 develop an immune response to this protein. The M protein of SARS-CoV (2002) also was the target of weak cross-interactions for these antibodies.

Ouyang J. et al., summarizing the literature data, reported that titers of neutralizing antibodies in the range of 1:40–1:640 are detected in patients with COVID-19 and that the titers for donor plasma should be at least 1:80–1:160. The same authors emphasized that high titers of antibodies do not prevent a severe case of COVID-19 [14]. The results of serological testing using the recombinant S and N proteins showed that the majority of patients develop stable antibody responses to these structural viral proteins between the 17th and 23rd days after the disease started. A slower but stronger antibody response has been observed in severe cases. It was noted that in patients with more severe cases of COVID-19, antibodies specific to the S protein showed high titers [15]. 

According to our data, it is shown that the presence of antibodies specific to RBD mostly correlates with the data on the neutralizing activity of blood sera. With almost all deaths, barring a few exceptions, the titers of neutralizing antibodies were at the level of severely ill convalescents and even higher, but this did not save this group of patients. Most likely, this happened, as described in the literature, due to multiple organ disorders in the infected body as a result of SARS-CoV-2’s immunopathogenesis. This was expressed in the fact that viral activation of dendritic cells and macrophages in lymphoid tissues leads to an excessive uncontrolled anti-inflammatory response, the so-called cytokine storm [16]. In addition, data have already been obtained on the dependence of the severity of COVID-19, which was established at the beginning of the pandemic, with increased IgG titers when compared with a low level of antibodies of this class in convalescents. The mechanism responsible for the immunopathological IgG response remains unclear [17].

It is not known whether patients from our random sample had previous contact with other seasonal coronaviruses (CoVs), for which antibodies are widely present in the human population. Several conserved regions have already been identified in the S2 domains of four known CoVs, namely 229E, NL63, OC43, HKUI, and SARS-CoV-2 [18], which is believed to be the cause of the antibody-dependent increase in the infection [19]. However, under ELISA and immunoblotting, when using the N recombinant protein of SARS-CoV-2 obtained in *E. coli*, cross-activity with this protein was observed only for antibodies of blood sera of patients who had previously recovered from SARS-CoV, but not with other CoVs [20]. In addition, the authors analyzed the amino acid sequence (aa) of the N protein of known CoVs isolated from humans. The N protein of SARS-CoV-2 turned out to have a homology of 19.1, 20.0, 26.5, 27.6, 46.1, and 90.5% with 229E, NL63, OC43, HKUI, MERS-CoV, and SARS-CoV, respectively [20].

In this study, we did not take into account the comorbidities of the patients. This aspect is also important for understanding viral pathogenesis. It is known that risk factors for a fatal outcome in COVID-19, in addition to male sex and old age [12], include diabetes mellitus and hyperglycemia that develops when infected with SARS-CoV-2 [21], cardiovascular diseases [22], etc. The analysis of the neutralizing activity of specific antibodies is an important stage in the study of the immune response of patients with COVID-19. Therefore, an extensive study of the immune response to SARS-CoV-2 in the population must be associated with such data for each patient with COVID-19, and this shows a further necessity for deeper investigations into the role of humoral immunity in COVID-19 disease. 

A varied course of coronavirus infections was observed over the course of a year and a half of fighting the pandemic. In this study, we highlighted the immune response in patients with a different course of COVID-19 at the very beginning of the pandemic in one of the major cities of Russia under restrictions (the absence of air and railway connections with neighboring countries). It may reflect the situation at a specific point and is valuable precisely in a retrospective analysis of the development of the epidemic process. We tried to get closer to understanding the role of humoral immunity in the development of pathogenesis, which still requires careful study.

## 4. Materials and Methods

### 4.1. Viral Preparations 

SARS-CoV-2/human/RUS/Nsk-FRCFTM-1/2020 strain was isolated from a clinical sample of a nasopharyngeal swab from the patient in Novosibirsk. The virus was purified and concentrated, as described [12]. Both SARS-CoV-2/human/RUS/Nsk-FRCFTM-1/2020 and an antigen of the SARS-CoV strain Frankfurt 1 (2002) [23] were stored at the Federal Research Center of Fundamental and Translational Medicine SB RAS at −80 °C. Viral preparations with an infectious titer of 6 lg tissue cytopathic doses (TCPD_50_/_mL_) were inactivated (in a 1:1 ratio) in a lysis buffer used for electrophoresis (composition for 2 mL: 1 M Tris-HCl with pH 6.8–0.5 mL; 10% sodium dodecyl sulfate (SDS) solution—0.8 mL; glycerin—0.2 mL; mercaptoethanol—0.2 mL; 0.4% Bromphenol Blue Na-sulf—0.1 mL). 

### 4.2. Recombinant Proteins 

On the basis of the nucleotide sequences of SARS-CoV-2 presented in GenBank: MN908947 [24], the plasmids for the expression of the full N and full S trimer (spike) and also its part in the S1 region, RBD, were constructed. Recombinant proteins of the S trimer and RBD were obtained in eukaryotic cells lines of female hamster ovarian (CHO-K1), and the nucleoproteins were obtained in *E. coli*. All recombinant proteins were purified with affinity chromatography.

### 4.3. Blood Sera

In this study, we utilized: 1. the convalescents’ blood sera from the employees of the Federal Research Center of the Fundamental and Translational Medicine SB RAS and their relatives who had suffered from mild cases of COVID-19, including asymptomatic cases, as well as moderate COVID-19 cases with pneumonia; 2. blood sera from intensive care unit patients with severe COVID-19, which were retrieved from the Infectious Diseases Clinical Hospital No. 1 in Novosibirsk. The diagnosis of COVID-19 in patients was based on the presence of SARS-CoV-2 RNA, which was detected using the RealBest SARS-CoV-2 RNA kit (Vector-Best, Novosibirsk, Russia) in a certified laboratory for the diagnosis of COVID-19 at the Federal Research Center of the Fundamental and Translational Medicine SB RAS. Permission to use the clinical material was obtained from the Ethics Committee of the Research Center (Protocol No. 17 as of 17 June 2020). The blood sera of convalescents and patients from the Infectious Disease Hospital were tested for the absence of SARS-CoV-2 RNA before conducting this study.

### 4.4. ELISA

IgG specific to SARS-CoV-2 was found using the author’s laboratory test system. Polystyrene plates (Nunk) were used for ELISA. The wells of plates were sensitized with both SARS-CoV-2 and SARS-CoV (strain Frankfurt 1, 2002) viral proteins and recombinant antigens at a concentration of 2 μg/mL in 100 μL of 0.05 M sodium phosphate buffer solution (pH 8.0) at 220 °C for 18 h. Nonspecific binding sites were saturated with 1% casein solution (Sigma, St. Louis, MO, USA) in TBST (Tris Buffered Saline with Tween containing 0.15 M NaCl; 0.02 M Tris-HCl pH 7.4; 0.05% Tween-20) within 45 min at 37 °C. Then, the antigens were incubated with blood sera (with their preliminary depletion of the nonspecific background by 5% of the volume of cell lysates, on which culture the corresponding antigens were produced) with a dilution of 1/100 (double step for titration) in a 0.5% casein solution for 1 h at 37 °C. The peroxidase conjugate of anti-species antibodies (Gout anti-human IgG, Sigma) was used at a working dilution of 1/6000 in 0.5% casein solution with incubation for 1 h at 37 °C. The immune response was manifested using a liquid substrate based on TMB (3,3′, 5,5′-tetramethylbenzidine). The reaction was blocked by adding 100 μL of 1 N HCl to each well. The absorbency of the substrate–indicator mixture was measured on a Uniscan spectrophotometer at a wavelength of 450 nm. A lysate of uninfected Vero cells and the blood serum of healthy people were used as a negative control of the antigen. The results of ELISA detected in three repetitions in two independent experiments were calculated using median values. 

### 4.5. Immunoblotting 

The SARS-CoV-2 and SARS-CoV (strain Frankfurt 1, 2002) viral proteins were separated in one wide “pocket” by electrophoresis in a 10% polyacrylamide gel (PAGE) supplemented with sodium dodecyl sulfate (SDS) and transferred to a nitrocellulose membrane (Millipore) in equipment (Cole-Parmer, Vernon Hills, IL, USA) for incubating blotting membranes for 5 h at 50 V, in a 0.025 M Tris-HCl buffer containing 0.192 M glycine (pH 8.3) and 20% ethanol. The sites of nonspecific binding were saturated with a 1% casein solution for 1.5 h at 20–22 °C. The whole membrane containing viral proteins was cut into separate strips, then numbered and incubated in separate containers for 4 h at 20–22 °C, with human blood sera (dilution 1:100) in a TSBT buffer containing 0.5% casein. After being washed in the TSBT buffer, the membrane strips were treated with anti-species antibodies labeled with horseradish peroxidase (Gout anti-human IgG, Sigma) at a working dilution of 1/6000 in 0.5% casein solution for 2 h at 37 °C. Then, the membrane strips were washed with TSBT buffer and developed in a chromagen solution (1 mg/mL 3.3 diaminobenzidine tetrahydrochloride in 50 mM Tris-HCl buffer (pH 7.4) containing 0.145 M NaCl, 20% ethanol, and 0.03% hydrogen peroxide). The reaction was stopped by washing the membrane strips in TSBT buffer. The specific interaction of the antibodies with viral proteins was manifested by the brown color of the stripes.

### 4.6. Virus Neutralization 

Virus neutralization with antibodies was carried out in accordance with the generally accepted method, as described [25]. The titer of the infectious virus was expressed in TCPD_50_/_mL_ (tissue cytopathic dose of the virus causing a 50% cytopathic effect on the infected cells). It was found by titrating the viral preparation on the Vero cells monolayer (African green monkey kidney cell culture) grown in 96-well culture plates (Corning, Glendale, AZ, USA). Before use, blood serum was inactivated by heating at 56 °C for 30 min to inactivate the antiviral effect of complement proteins. Before applying it to a monolayer of cells, blood serum ranging from 1 to 20 with two dilutions was preincubated with the infectious SARS-CoV-2/human/RUS/Nsk-FRCFTM-1/2020 strain in a titer of 103 TCPD_50_/mL (50% tissue cytopathic doses in mL) for 1 h at 37 °C in a nutrient medium containing a 2% blood serum (heated at 56 °C for 30 min) of cattle. Then, it was applied to a monolayer of cell culture in three replicates. After the incubation of the mixture of antibodies with the virus for 1 h at 37 °C, the monolayer of cells was washed and left in a nutrient medium containing 2% cattle blood sera until a cytopathogenic effect was observed in control wells containing infected cells. To observe the infected and control cells in dynamics, an inverted microscope Mikromed I (Mikromed, Sankt Petersburg, Russia) was used at 10× magnification. The cells were fixed for 30 minutes with a formaldehyde solution and a 0.05% crystalline violet solution with 20% alcohol, as described [26]. Then, the liquid from the wells was removed and washed with water. The results were recorded visually. The neutralizing antibodies titer was considered the final dilution of blood serum at which cells were protected from the cytopathogenic effect in 50% of the wells. The neutralizing antibodies titer, which was detected in three repetitions in two independent experiments, was calculated using the median results.

## Data Availability

The data presented in this study are available in the main text, figures, tables and supplementary material.

## Supplementary Materials

The following are available online at https://www.mdpi.com/article/10.3390/pathogens10111421/s1, Table S1: Specific interaction of inactivated viruses and recombinant proteins in IgG ELISA of convalescents and patients died from COVID-19; Table S2: The neutralizing activity of the blood serum of convalescents and patients with COVID-19; Table S3. The neutralizing activity of the blood serum of patients who subsequently died in hospital from COVID-19.

## References

[1] WHO Report World Health Organization. WHO Coronavirus Disease (COVID-19) Dashboard. https://www.who.int/emergencies/diseases/novel-coronavirus-2019.

[2] Chou R., Dana T., Buckley D.I., Selph S., Fu R., Totten A.M. (2020). Epidemiology of and Risk Factors for Coronavirus Infection in Health Care Workers. Ann. Intern. Med..

[3] Sanche S., Lin Y.T., Xu C., Romero-Severson E., Hengartner N., Ke R. (2020). High Contagiousness and Rapid Spread of Severe Acute Respiratory Syndrome Coronavirus 2. Emerg. Infect. Dis..

[4] WHO Draft Landscape and Tracker of COVID-19 Candidate Vaccines. https://www.who.int/publications/m/item/draft-landscape-of-covid-19-candidate-vaccines.

[5] Ni L., Ye F., Cheng M.-L., Feng Y., Deng Y.-Q., Zhao H., Wei P., Ge J., Gou M., Li X. (2020). Detection of SARS-CoV-2-Specific Humoral and Cellular Immunity in COVID-19 Convalescent Individuals. Immunity.

[6] Jeyanathan M., Afkhami S., Smaill F., Miller M., Lichty B.D., Xing Z. (2020). Immunological considerations for COVID-19 vaccine strategies. Nat. Rev. Immunol..

[7] Cyпотницкий M.B. (2020). COVID-19 Pandemic as an Indicator of «Blank Spots» in Epidemiology and Infectious Pathology. J. NBC Prot. Corps.

[8] O’Driscoll M., Dos Santos G.R., Wang L., Cummings D.A.T., Azman A.S., Paireau J., Fontanet A., Cauchemez S., Salje H. (2020). Age-specific mortality and immunity patterns of SARS-CoV-2. Nature.

[9] Huang C., Wang Y., Li X., Ren L., Zhao J., Hu Y., Zhang L., Fan G., Xu J., Gu X. (2020). Clinical features of patients infected with 2019 novel coronavirus in Wuhan, China. Lancet.

[10] Qiu M., Shi Y., Guo Z., Chen Z., He J., Chen R., Zhou D., Dai E., Wang X., Si B. (2005). Antibody responses to individual proteins of SARS coronavirus and their neutralization activities. Microbes Infect..

[11] Guan M., Chen H.Y., Tan P.H., Shen S., Goh P.-Y., Tan Y.-J., Pang P.H., Lu Y., Fong P.Y., Chin D. (2004). Use of Viral Lysate Antigen Combined with Recombinant Protein in Western Immunoblot Assay as Confirmatory Test for Serodiagnosis of Severe Acute Respiratory Syndrome. Clin. Vaccine Immunol..

[12] Chepurnov A.A., Sharshov K.A., Kazachinskaya E.I., Kononova Y.V., Kazachkova E.A., Khripko O.P., Yurchenko K.S., Alekseev A.Y., Voevoda M.I., Shestopalov A.M. (2020). Antigenic properties of sARs-CoV-2/human/RUs/nsk-FRCFtM-1/2020 coronavirus isolate from a patient in novosibirsk. J. Infectology.

[13] Sturman L.S. (1977). Characterization of a coronavirus. Virology.

[14] Ouyang J., Isnard S., Lin J., Fombuena B., Peng X., Routy J.-P., Chen Y. (2020). Convalescent Plasma: The Relay Baton in the Race for Coronavirus Disease 2019 Treatment. Front. Immunol..

[15] Qu J., Wu C., Li X., Zhang G., Jiang Z., Li X., Zhu Q., Liu L. (2020). Profile of Immunoglobulin G and IgM Antibodies Against Severe Acute Respiratory Syndrome Coronavirus 2 (SARS-CoV-2). Clin. Infect. Dis..

[16] Zhou G., Zhao Q. (2020). Perspectives on therapeutic neutralizing antibodies against the Novel Coronavirus SARS-CoV-2. Int. J. Biol. Sci..

[17] Zhang B., Zhou X., Zhu C., Song Y., Feng F., Qiu Y., Feng J., Jia Q., Song Q., Zhu B. (2020). Immune Phenotyping Based on the Neutrophil-to-Lymphocyte Ratio and IgG Level Predicts Disease Severity and Outcome for Patients With COVID-19. Front. Mol. Biosci..

[18] Lan J., Ge J., Yu J., Shan S., Zhou H., Fan S., Zhang Q., Shi X., Wang Q., Zhang L. (2020). Structure of the SARS-CoV-2 spike receptor-binding domain bound to the ACE2 receptor. Nature.

[19] Tetro J.A. (2020). Is COVID-19 receiving ADE from other coronaviruses?. Microbes Infect..

[20] Guo L., Ren L., Yang S., Xiao M., Chang D., Yang F., Cruz C.S.D., Wang Y., Wu C., Xiao Y. (2020). Profiling Early Humoral Response to Diagnose Novel Coronavirus Disease (COVID-19). Clin. Infect. Dis..

[21] Lim S., Bae J.H., Kwon H.-S., Nauck M.A. (2020). COVID-19 and diabetes mellitus: From pathophysiology to clinical management. Nat. Rev. Endocrinol..

[22] Azevedo R.B., Botelho B.G., De Hollanda J.V.G., Ferreira L.V.L., De Andrade L.Z.J., Oei S.S.M.L., Mello T.D.S., Muxfeldt E.S. (2020). Covid-19 and the cardiovascular system: A comprehensive review. J. Hum. Hypertens..

[23] Agafonov A.P., Gus’Kov A.A., Ternovoi V.A., Ryabchikova E.I., Durymanov A.G., Vinogradov I.V., Maksimov N.L., Ignat’Ev G.M., Nechaeva E.A., Netesov C.M.O.R.S.V. (2004). Primary Characterization of SARS Coronavirus Strain Frankfurt 1. Dokl. Biol. Sci..

[24] Wu F., Zhao S., Yu B., Chen Y.-M., Wang W., Song Z.-G., Hu Y., Tao Z.-W., Tian J.-H., Pei Y.-Y. (2020). A new coronavirus associated with human respiratory disease in China. Nature.

[25] Van Doremalen N., Lambe T., Spencer A., Belij-Rammerstorfer S., Purushotham J.N., Port J.R., Avanzato V.A., Bushmaker T., Flaxman A., Ulaszewska M. (2020). ChAdOx1 nCoV-19 vaccine prevents SARS-CoV-2 pneumonia in rhesus macaques. Nature.

[26] Case J.B., Bailey A.L., Kim A.S., Chen R.E., Diamond M.S. (2020). Growth, detection, quantification, and inactivation of SARS-CoV-2. Virology.

