# Supplementary material for: IgG Study of Blood Sera of Patients with COVID-19
Source: Pathogens. 2021 Nov 2;10(11):1421. doi: 10.3390/pathogens10111421 (PMC8621046; doi:10.3390/pathogens10111421)
Supplement: Supplementary file 1 [file pathogens-10-01421-s001.zip › Table S1. Specific interaction of inactivated viruses and recombinant proteins in IgG ELISA.pdf]

Table S1. Specific interaction of inactivated viruses and recombinant proteins in IgG ELISA of convalescents and patients died from COVID-19

No.	Antigens and titers of antibody specificity (median/mean±SD)									
	RBD		full S trimer		full N		SARS-CoV-2		SARS-CoV (2002)	
1	800	1066,67±330,56	3200	4266,66±1322,23	6400	8533,33±2644,46	6400	5333,33±1322,23	800	1066,67±330,56
2	200	233,33±65,33	200	266,67±82,64	800	666,67±165,28	200	266,67±82,64	100	116,67±32,67
3	400	466,67±130,66	400	466,67±130,66	1600	2133,33±661,11	800	933,33±261,33	200	233,33±65,33
4	200	266,67±82,64	400	466,67±130,66	1600	2133,33±661,11	800	733,33±130,66	200	233,33±65,33
5	800	733,33±130,66	800	733,33±130,66	1600	1733,33±629,36	1600	1333,33±330,56	400	533,33±165,28
6	800	933,33±261,33	800	933,33±261,33	3200	2666,67±661,11	1600	2133,33±661,11	400	466,67±130,66
7	800	1066,67±330,56	1600	2133,33±661,11	3200	2666,67±661,11	3200	2933,33±522,66	800	733,33±130,66
8	400	533,33±165,28	800	1066,67±330,56	1600	2133,33±661,11	3200	2666,67±661,11	1600	1333,33±330,56
9	1600	1333,33±330,56	3200	2933,33±522,66	6400	8533,33±2644,46	3200	3733,33±1045,31	1600	1866,67±522,66
10	400	533,33±165,28	1600	1466,67±261,33	3200	4266,66±1322,23	3200	4266,66±1322,23	800	1066,67±330,56
11	800	933,33±261,33	1600	1466,67±261,33	3200	3733,33±1045,31	3200	4266,66±1322,23	800	933,33±261,33
12	400	466,67±130,66	1600	2133,33±661,11	6400	7466,67±2090,63	3200	3733,33±1045,31	800	933,33±261,33
13	800	1066,67±330,56	800	1066,67±330,56	1600	1466,67±261,33	800	1066,67±330,56	200	233,33±65,33
14	1600	1333,33±330,56	6400	5333,33±1322,23	3200	3200,00±1402,44	3200	2666,67±661,11	400	533,33±165,28
15	1600	1466,67±261,33	3200	4266±1322,23	6400	7466,67±2090,63	6400	5333,33±1322,23	1600	1333,33±330,56
16	3200	2933,33±522,66	6400	5866,67±1045,31	12800	10666,67±6201,80	12800	10666,67±6201,80	3200	2666,67±661,11
17	200	233,33±65,33	400	533,33±165,28	800	1066,67±330,56	400	533,33±165,28	100	116,67±32,67
18	1600	1733,33±629,36	1600	1733,33±629,36	3200	4266,66±1322,23	3200	2933,33±522,66	1600	1466,67±261,33
19	1600	1333,33±330,56	3200	4266,66±1322,23	1600	2133,33±661,11	3200	3733,33±1045,31	800	933,33±261,33
20	3200	2666,67±661,11	3200	4266,66±1322,23	6400	5333,33±1322,23	6400	6933,33±2517,45	1600	1333,33±330,56
21	800	733,33±130,66	1600	2133,33±661,11	3200	4266,66±1322,23	3200	4266,67±1322,23	800	666,67±165,28
22	3200	3733,33±1045,31	6400	8533,33±2644,46	6400	5333,33±1322,23	6400	7466,67±2090,63	3200	2666,67±661,11
23	200	233,33±65,33	400	533,33±165,28	800	1333,33±775,23	400	533,33±165,28	100	133,33±41,319
24	1600	1333,33±330,56	6400	7466,67±2090,63	12800	10666,67±6201,80	6400	8533,33±2644,46	1600	1866,67±522,66
25	800	1066,67±330,56	1600	2000,00±783,98	3200	4266,66±1322,23	3200	4266,66±1322,23	400	366,66±65,33

26	1600	2133,33±661,11	6400	7466,67±2090,63	6400	5333,33±1322,23	6400	5333,33±1322,23	1600	1333,33±330,56
27	3200	2933,33±522,66	3200	4266,66±1322,23	3200	4266,66±1322,23	3200	4266,66±1322,23	800	1066,67±330,56
28	3200	3466,66±1258,72	6400	7466,67±2090,63	12800	10666,67±6201,80	6400	5333,33±1322,23	3200	2666,67±661,11
29	3200	4266,66±1322,23	6400	7466,67±2090,63	12800	13866,67±5034,99	12800	10666,67±6201,80	3200	3733,33±1045,31
30	3200	3733,33±1045,31	3200	4000,00±1567,97	12800	10666,67±6201,80	6400	8533,33±2644,46	1600	2133,33±661,11
31	1600	1733,33±629,36	1600	2133,33±661,11	12800	10666,67±6201,80	6400	7466,67±2090,63	1600	1733,33±629,36
32	3200	4266,66±1322,23	3200	4266,66±1322,23	12800	11733,33±2090,628	12800	10666,67±6201,80	6400	5333,33±1322,23
33	25600	21333,33±5288,92	25600	21333,33±5288,92	6400	8533,33±2644,46	25600	23466,67±4181,26	25600	23466,67±4181,26
34	1600	2133,33±661,11	6400	8533,33±2644,46	3200	4266,66±1322,23	12800	17066,67±5288,92	12800	17066,67±5288,92
35	3200	2933,33±522,66	12800	10666,67±6201,80	6400	8533,33±2644,46	12800	14933,33±4181,26	12800	17066,67±5288,92
36	100	133,33±41,32	200	266,67±82,64	100	133,33±41,32	400	533,33±165,28	400	533,33±165,28
37	200	266,67±82,64	800	1000,00±391,992	400	366,67±65,33	1600	1333,33±330,56	1600	1333,33±330,56
38	800	1066,67±330,56	3200	4266,66±1322,23	3200	4266,66±1322,23	12800	17066,67±5288,92	25600	21333,33±5288,92
39	1600	2133,33±661,11	6400	5333,33±1322,23	1600	2133,33±661,11	6400	5333,33±1322,23	12800	17066,67±5288,92
40	3200	4266,66±1322,23	6400	8533,33±2644,46	6400	7466,67±2090,63	12800	17066,67±5288,92	12800	17066,67±5288,92
41	6400	5333,33±1322,23	12800	10666,67±6201,80	12800	10666,67±6201,80	25600	21333,33±5288,92	25600	21333,33±5288,92
42	100	133,33±41,32	400	533,33±165,28	100	116,67±32,67	200	266,67±82,64	400	533,33±165,28
43	3200	4266,66±1322,23	3200	4266,66±1322,23	1600	2133,33±661,11	3200	4266,66±1322,23	6400	8533,33±2644,46
44	400	533,33±165,28	800	666,67±165,28	1600	2133,33±661,11	3200	4266,66±1322,23	6400	8533,33±2644,46
45	3200	4266,66±1322,23	3200	4266,66±1322,23	1600	1866,67±522,66	6400	7466,67±2090,63	12800	10666,67±6201,80
46	800	933,33±261,33	800	1000,00±391,992	400	533,33±165,28	1600	2133,33±661,11	3200	4266,66±1322,23
47	1600	2133,33±661,11	1600	2133,33±661,11	1600	1333,33±330,56	6400	8533,33±2644,46	12800	17066,67±5288,92
48	800	1066,67±330,56	6400	5333,33±1322,23	1600	2133,33±661,11	3200	3733,33±1045,31	3200	4266,66±1322,23
49	6400	5333,33±1322,23	25600	21333,33±5288,92	6400	8533,33±2644,46	12800	17066,67±5288,92	6400	8533,33±2644,46
50	400	533,33±165,28	1600	2666,67±1550,45	800	1066,67±330,56	1600	2133,33±661,11	6400	8533,33±2644,46
51	1600	2000,00±783,98	1600	2133,33±661,11	400	533,33±165,28	1600	1333,33±330,56	3200	4266,66±1322,23
52	1600	2133,33±661,11	3200	4266,66±1322,23	1600	1333,33±330,56	3200	4266,66±1322,23	6400	8533,33±2644,46
53	800	1066,67±330,56	800	1066,67±330,56	200	266,67±82,64	800	1066,67±330,56	1600	2133,33±661,11
54	3200	4266,66±1322,23	6400	8533,33±2644,46	1600	1333,33±330,56	3200	2666,67±1550,45	6400	5333,33±1322,23

Note. SD - standard deviations
