# Supplementary material for: IgG Study of Blood Sera of Patients with COVID-19
Source: Pathogens. 2021 Nov 2;10(11):1421. doi: 10.3390/pathogens10111421 (PMC8621046; doi:10.3390/pathogens10111421)
Supplement: Supplementary file 1 [file pathogens-10-01421-s001.zip › Table S2. The neutralizing activity of the blood serum_2.pdf]

Table S2. The titer of neutralizing antibodies was found at 10^3 $TCPD_{50}$/ml SARS-CoV-2. Blood sera: No. 1 – an asymptomatic case; No. 2-14 – mild cases, No. 15 – a moderate case with unilateral pneumonia; No. 16 – a moderate case with hospitalization for bilateral pneumonia; No. 17-32 - severe cases of the patients who were treated in the intensive care unit.

No.	Age	Sex	Neutralizing antibody titer
1	32	M	80
2	33	M	20
3	40	F	40
4	37	M	10
5	51	F	40
6	41	M	40
7	44	M	160
8	42	F	20
9	35	F	160
10	26	F	20
11	64	F	80
12	64	M	40
13	41	M	40
14	41	F	160
15	55	F	80
16	70	M	≥320
17	74	F	0
18			≥320
19	64	F	160
20			≥320
21	43	M	80
22			160
23	59	M	0
24			160
25	56	F	80
26			160
27	62	M	160
28			≥320
29	65	M	160
30	64	M	160
31	59	M	160
32	71	M	160
