# Supplementary material for: IgG Study of Blood Sera of Patients with COVID-19
Source: Pathogens. 2021 Nov 2;10(11):1421. doi: 10.3390/pathogens10111421 (PMC8621046; doi:10.3390/pathogens10111421)
Supplement: Supplementary file 1 [file pathogens-10-01421-s001.zip › Table S3. The neutralizing activity of the blood serum -2.pdf]

Table S3. The titer of neutralizing antibodies was found at 10^3 $TCPD_{50}$/ml SARS-CoV-2. Note: time of blood sampling (in days) before death.

No.	Age	Sex	Days before death	Neutralizing antibody titer
33	71	F	3	≥320
34	65	M	1	160
35	71	M	1	≥320
36	92	M	1	0
37	79	F	7	20
38			2	80
39	65	M	15	160
40			10	≥320
41			7	≥320
42	59	F	18	0
43			2	160
44	91	M	13	40
45			9	80
46	59	M	13	80
47			8	160
48	73	F	19	80
49			12	≥320
50	91	M	18	20
51	63	F	12	160
52			10	160
53	63	F	21	160
54			5	≥320
